# Changes of inequality in functional disability of older populations in China from 2008 to 2018: a decomposition analysis

**DOI:** 10.1186/s12877-022-02987-8

**Published:** 2022-04-09

**Authors:** Tao Zhang, Chaojie Liu, Beiyin Lu, Xiaohe Wang

**Affiliations:** 1 Department of Health Policy and Management, School of Public Health, Hangzhou Normal University, No. 2318, Yuhangtang Rd., Zhejiang, 311121 Hangzhou China; 2 School of Psychology and Public Health, La Trobe University, Melbourne, Australia

**Keywords:** Functional disability; inequality, Older adults, Oaxaca decomposition, China

## Abstract

**Background:**

This study aims to determine the change of inequality in functional disability of older populations in China over the period from 2008 to 2018 and decompose the contribution of the personal and environmental predictors to the change.

**Methods:**

Data were drawn from two waves (2008 and 2018) of the Chinese Longitudinal Healthy Longevity Survey (CLHLS). Functional disability was assessed by the basic activities of daily living (ADL) and the instrumental activities of daily living (IADL). Concentration index (CI) was calculated to measure the socioeconomic inequality in ADL and IADL. A two-level linear regression model was established to identify the individual and care environmental predictors and their contribution to the inequality of ADL and IADL, respectively. The Oaxaca-type decomposition technique was adopted to estimate the contribution of these predictors to changes of the inequality in ADL and IADL over the period from 2008 to 2018.

**Results:**

Socioeconomic inequality in functional disability of older adults increased over the period from 2008 to 2018, with the CI for ADL changing from − 0.0085 to − 0.0137 and the CI for IADL changing from − 0.0164 to − 0.0276, respectively. Self-rated economic status was the single most powerful predictor of changes in the inequality, although the growing and dominant rating of older persons with fare economic status could offset the detrimental effects of other (rich or poor) ratings on the changes. The enlarged inequality was also attributable to the increasing importance of regular exercise and its distributional changes, as well as the accumulative long-term effect of farming in earlier life. They outweighed the counteracting effects of rural residency, living with chronic conditions and in an institution.

**Conclusions:**

Socioeconomic inequality in functional disability of older populations in China increased over the period from 2008 to 2018. Re-distribution of wealth remains to be a powerful instrument for addressing the inequality issue, but alone it is not enough. The detrimental accumulative effect of farming will not disappear any time soon. While rural residents are catching up with their urban counterparts, new challenges such as physical inactivity are emerging.

**Supplementary Information:**

The online version contains supplementary material available at 10.1186/s12877-022-02987-8.

## Background

Health inequity is a major concern in public policy interventions and an essential indicator for assessing health system performance. Paradoxically, the growing number of older populations as a result of good health system performance imposes a great challenge for maintaining health equality [1]. Older age is usually associated with decreased socioeconomic status (SES). Meanwhile, the social and health inequalities experienced by the disadvantaged accumulate over their life course [2]. Significant socioeconomic inequalities in both physical and mental health have been observed in the older populations in both developing and developed countries [3–5]. China is no exception.

A large body of literature consistently reported low SES in people with disability as measured by the Global Activity Limitation Indicator (GALI) and the Activities of Daily Living (ADL). For example, the populations with a higher prevalence of disability often experience lower school attendance, higher unemployment rate, and lower income [3, 4, 6–8]. Their poor health status is likely to be exacerbated by poor knowledge and low affordability of health services. With the increase of life expectancy, more people are living with disability and health inequality tends to increase rather than decrease [6].

China has the largest older population in the world. This is not only because China is the most populated country with over 1.4 billion people, but also because it has experienced a rapid ageing process as a result of increased life expectancy and declined fertility [9]. Consequently, the populations living with disability have increased dramatically in China. It was estimated that about 16% of old adults (≥60 years) in China have experienced limitations in at least one of the daily activities (eating, dressing, getting in and out of bed, going to the toilet, walking indoors, bathing). This amounts to over 42 million people living with disability [10], leading to significant burdens in medical care, social services, and long-term care [11].

Socioeconomic inequality of disability in later life is persistent and large in China [5, 12]. It appears that the rural older are most likely to live with disability in China compared with other populations [12–14]. Rahman et al. [15] compared the socioeconomic inequality of disability in older adults across the low- and middle-income countries (LMICs), and China was ranked on top with the highest inequality. Kuma et al. [16] found that income inequality is most profound between the older adults living with and without disability in China.

The Chinese government has launched a series of policies to address the issue of social and health inequalities since 2009, which include the establishment of universal coverage of social security programs, such as social health insurance and aged pension insurance [17, 18]. There is clear evidence that the socioeconomic disadvantaged populations have obtained benefits from these initiatives [17, 19]. However, because financial co-contributions are required for enjoying the insurance-alike entitlements, some researchers argued that the well-off populations may benefit more than those worst-off [20]. This could potentially increase the socioeconomic inequality.

Extensive studies have been conducted to generate evidence about the socioeconomic inequality in disability of older people in China [12–16]. However, there is paucity in the literature documenting changes in the inequality, let alone the contributing factors for the changes (if any). This study aimed to address the gap in the literature by answering the following two questions: (1) What had been changed from 2008 to 2018 in terms of the socioeconomic inequality in disability of older people in China? (2) What personal and environmental factors had contributed to the changes, if any?

## Methods

### Data source

Data used in this study were extracted from the Chinese Longitudinal Healthy Longevity Survey (CLHLS). The CLHLS monitored the health and (biological, behavioural and social) health risk factors of older adults (≥65 years) populations in China. It has been conducted every two or three years since 1998 using a multistage stratified random cluster sampling strategy. The survey sample was drawn from almost half counties/cities of 23 provinces that covered about 85% of China’s entire population [21, 22]. Only one older member from each participating household was invited to participate in the survey. The selection of participants considered a balance of gender and age, with approximately equal numbers of male and female participants across the young-old (65–79 years), octogenarians (80–89 years) and nonagenarians (90–99 years) groups. Details of the survey protocol can be found in previous publications [21, 22].

In total, eight waves of surveys have been completed so far. Two waves of data ten years apart were used for the purpose of this study. We compared the inequality in functional disability of the study participants in the most recent survey (*n* = 15,874) in 2018 with those in the 2008 survey (*n* = 16,954). After excluding the records containing missing values in the variables of interest, a final sample of 13,551 (80%) for the 2008 cohort and 13,514 (85%) for the 2018 cohort were included in data analyses. There were 282 participants who participated in both surveys. In order to reduce calculation bias, we excluded these repeated respondents in 2018 wave (*n* = 13,232) for final data analysis.

### Measurements

#### Outcome variables

Functional disability was measured by two variables: basic activities of daily living (ADL) and instrumental activities of daily living (IADL). The ADL assesses self-reported needs for assistance in eating, dressing, indoor mobility, bathing, toileting, and continence. Study participants were asked to rate each item on a three-point scale, ranging from 1 ‘no assistance needed’ to 3 ‘full assistance needed’. A summed score (range 6–18) was calculated, with a higher score indicating higher dependence [23, 24]. The IADL assesses ability to do cooking, laundry, walking for two kilometers without stopping, lifting up to 10 kg, repeated squatting and standing, using public transport alone, shopping, and socialising. Study participants were asked to rate their ability against each task on a three-point scale, ranging from 1 ‘no difficulty’ to 3 ‘cannot do’. A summed score was calculated (range 8–24), with a higher score indicating a higher level of functional disability [23, 24].

#### Socioeconomic status

Inequalities of the ADL and IADL scores were estimated in the study participants with different self-rated economic status. The study participants were asked to rate their economic status in comparison with others in their local region on a five-point Likert scale, ranging from 1 “very poor” to 5 “very rich”. The self-rated indicator was chosen for several reasons. Firstly, there were great regional disparities in living standards and pricing of consumptions in China and household income in absolute terms would not fully reflect SES. Secondly, it was challenging to accurately estimate household income when multiple sources of income existed, and a recall bias was inevitable especially in older adults. Thirdly, self-rated economic status represents a rating in comparison with the local residents, which is an indication of influence of a wide array of socioeconomic factors such as income, living condition, consumption level, and occupation. Self-reported SES has been found to have stronger associations with both physical and mental health compared with the objective SES indicators [25, 26].

### Explanatory variables

**Fig. 1:**
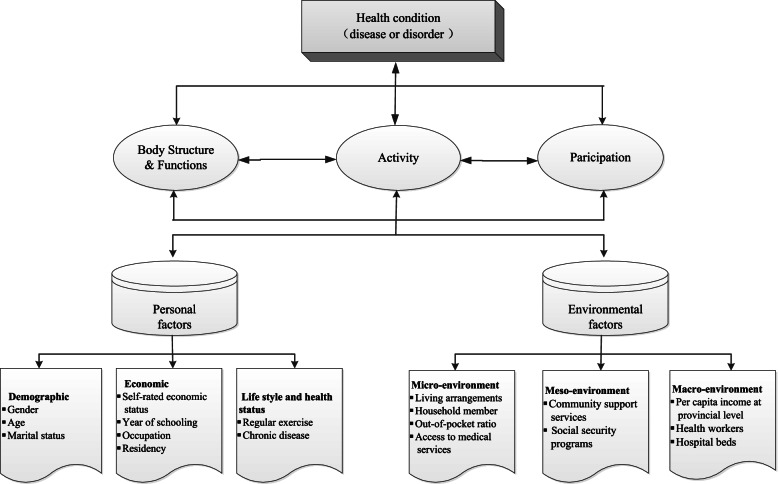
Personal and environmental explanatory variables of functional disability

Limitations in ADL and IADL are determined by multiple factors. The International Classification of Functioning, Disability and Health (ICF) framework categories these factors into personal and environmental [27, 28]. The personal factor covers the biological and behavioural characteristics of a person, such as age, sex, education and lifestyle. The environmental factor extends from the immediate environment in which a person is living, studying and working to the broad society defined by its physical, social and cultural environments (Fig. 1).

In this study, the available data were mapped into the ICF framework (Fig. 1). Two-levels of measurements were identified as explanatory variables. The demographic (sex, age and marital status), socioeconomic (self-rated economic status, years of schooling, occupation and residency), and lifestyle (regular exercise) characteristics of the study participants, and their health (chronic diseases), micro-environmental (living arrangements, household member living together, access to medical services, and out-of-pocket payment ratio for medical bills) and meso-environmental (community support services available and social security programs covered) indicators served as the individual-level measurements. The macro-environmental (income per capita at the provincial level, skilled health workers and hospital beds per 10,000 people) indicators served as the provincial-level measurements, which were obtained from the provincial health statistics reports. Details about the definition of these measurements are provided in the supplementary file (Table S1 in supplementary file).

### Statistical analysis

#### Measuring inequality

Concentration index (CI) was calculated to measure socioeconomic inequality of functional disability. It is twice the (weighted) covariance of the ADL (or IADL) scores (*y*) and the relative rank of the study participants in their self-rated economic status (*γ*), divided by the mean of the ADL (or IADL) scores [29, 30].

$$\mathrm{CI}=\frac{2}{\mu } COV\left(y,\gamma \right)$$

The value of CI ranges between -1 and + 1. A value of zero indicates an absence of inequality, while a greater distance from zero indicates a higher level of inequality. The negative sign means that those with a lower SES tend to suffer more from functional disability, and vice versa.

#### Decomposing inequality

The contribution of the explanatory variables to the CI was decomposed using the method proposed by Wagstaff et al. [31]. A two-level linear regression model was established for the ADL and IADL scores (*y*), respectively, with the first-level (*k refers to individual level*) and second-level (*m refers to provincial level)* predictors (*x*).

$${y}_i={a}^m+{\sum}_k{\beta}_k^m{x}_{ki}+{\upvarepsilon}_i$$

Where *i* represents each individual participant; ${\beta}_k^m$ is the marginal effect of each predictor x; *Ɛ*_*i*_ represents the error term.

The CI of the ADL or IADL scores (*y*) can be expressed as:

$$\mathrm{CI}={\sum}_k\left({\beta}_k^m\overline{x_k}/\mu \right){c}_k+{GC}_{\varepsilon }/\mu$$

Where $\overline{x_k}\ {c}_k$ is the normalised concentration index of *x*_*k*_; *GC*_*ε*_ is the generalised concentration index for the error term (ε); μ is the mean value of the ADL or IADL scores (*y*). This equation reveals two components of CI: the first is explained by elasticity (${\beta}_k^m\overline{x_k}/\mu$), a unit-free measure indicating changes in the concentration index for the ADL or IADL scores as a result of one-unit change in the explanatory variable; the second is residual, the component (*GC*_*ε*_/*μ*) that cannot be explained by the predictors.

#### Decomposing changes in inequality

The contribution of each predictor to the changes of the CI of the ADL or IADL scores (∆C) over the period from 2008 to 2018 was determined using the Oaxaca-type decomposition method [31, 32].

$$\Delta \mathrm{C}={\sum}_k{\eta}_{kt}\left({c}_{kt}-{c}_{kt-1}\right)+{\sum}_k{c}_{kt-1}\left({\eta}_{kt}-{\eta}_{kt-1}\right)+\Delta \left({GC}_{\varepsilon t}\left/ {\mu}_t\right.\right)$$

Where *η*_*kt*_ and *η*_*kt* − 1_ represent the elasticity of the explanatory variable in the year of 2018 and 2008, respectively, while *c*_*kt*_ and *c*_*kt* − 1_ indicate the normalised CI of the explanatory variable in 2018 and 2008, respectively. $\Delta \left({GC}_{\varepsilon t}\left/ {\mu}_t\right.\right)$ is the change of residual over time. The changes in inequality can be attributed to two changes: (1) distributional change (Δc*η_k_) as a result of distribution differences of the explanatory variables in different years; and (2) elasticity change (Δη*c_kt-1_) as a result of differential responses of the outcome variable to the explanatory variables in different years.

All statistical analyses were performed using STATA 14.0. All methods were performed in accordance with the relevant guidelines and regulations.

## Results

### Characteristics of study participants

In both waves, most participants were older than 80 years. More than half of the respondents are women. 68.17 and 71.07% of people rated their economic status as fare in 2008 and 2018, respectively. More than half did not attend school at all and were engaged in farming in their earlier life. Less than 30% reported regular exercise. More than 60% had chronic conditions. The vast majority (> 80%) lived with their family members (Table 1).

**Table 1:** Characteristics of study participants in 2008 and 2018

Characteristics	2008 (***n*** = 13,551)		2018 (***n*** = 13,232)		*p*
	N	%	N	%	
**Sex**					0.001
Male	5637	41.59	5769	43.59	
Female	7914	58.41	7463	56.40	
**Age (Years)**					< 0.001
≤ 80	3805	28.07	4815	36.38	
> 80	9746	71.93	8417	63.61	
**Marital status**					< 0.001
With spouse	4031	29.75	5226	39.49	
Others	9520	70.25	8006	60.50	
**Self-rated economic status**					< 0.001
Very poor	432	3.18	181	1.36	
Poor	2115	15.60	1108	8.37	
Fair	9238	68.17	9404	71.07	
Rich	1638	12.08	2234	16.88	
Very rich	128	0.94	305	2.30	
**Years of schooling**					< 0.001
0	8582	63.33	7617	57.56	
1–5	2928	21.60	2555	19.30	
≥ 6	2041	15.07	3060	23.12	
**Occupation**					< 0.001
Agriculture	9040	66.71	7052	53.29	
Others	4511	33.29	6180	46.71	
**Regular exercise**					< 0.001
Yes	3671	27.09	3897	29.45	
No	9880	72.91	9335	70.54	
**Chronic disease**					< 0.001
No	5276	38.93	4273	32.29	
Yes	8275	61.06	8959	67.70	
**Residency**					< 0.001
Urban	5361	39.56	7482	56.54	
Rural	8190	60.44	5750	43.45	
**Community support services**					< 0.001
0	9694	71.54	4798	36.26	
≥ 1	3857	28.46	8434	63.73	
**Social security program enrolment**					< 0.001
0	3111	22.95	884	6.68	
1	7363	54.33	6673	50.43	
≥ 2	3077	22.71	5675	42.88	
**Living arrangement**					< 0.001
With family members	11,295	83.35	10,804	81.65	
Alone	2034	15.01	2022	15.28	
In an institution	222	1.64	406	3.06	
**Household members**					< 0.001
0	2870	16.90	2428	18.35	
1–2	8013	47.30	7012	52.99	
≥ 3	6069	38.80	3792	28.65	
**Access to medical care**					< 0.001
Yes	12,569	92.75	12,924	97.67	
No	982	7.25	308	2.33	
**Out-of-pocket payment ratio (%) for medical bills (Mean ± SD**^**a**^**)**	86.87 ± 27.96		73.24 ± 32.08		< 0.001
**Per capita income at the provincial level (Mean ± SD)**	9724.01 ± 6431.31		31,050.07 ± 11,649.08		< 0.001
**Skilled health workers per 1000 people (Mean ± SD)**	4.00 ± 1.68		7.05 ± 1.18		< 0.001
**Hospital beds per 1000 people (Mean ± SD)**	3.13 ± 1.05		6.00 ± 0.75		< 0.001

^a^ *SD* standard deviation

The 2018 respondents were younger, more likely to attend school, to live with a spouse and to do regular exercise, and less likely to report poor or very poor economic status, to engage in farming, and to live without a chronic condition compared with their 2008 counterparts. About 56% of the 2018 respondents lived in urban areas, compared with 40% in 2008. The 2018 respondents enjoyed more community support, social security entitlements, and medical services than their 2008 counterpart, albeit sharing a lower proportion of medical bills out-of-pocket. The health resources in terms of skilled health workers and hospital beds per 1000 population in 2018 were more than 1.7 times of those in 2008 (Table 1).

### Inequality of functional disability

The average ADL score increased from 7.09 (SD = 2.50) in 2008 to 7.65 (SD = 2.83) in 2018. Similarly, the average IADL score increased from 13.22 (SD = 6.41) to 14.87 (SD = 6.07). Both changes were statistically significant (*p* < 0.001).

The inequality of functional disability also increased, with the CI value changing from − 0.0085 in 2008 to − 0.0141 in 2018 for ADL, and from − 0.0164 in 2008 to − 0.0279 in 2018 for IADL. The results indicate that the poor were more likely to suffer from functional disability and the gap between the poor and the rich was enlarged over the ten-year period.

### Decomposition of inequality

The major contributors to the inequality varied for the two functional disability indicators (ADL and IADL) and between 2008 and 2018. In 2008, out-of-pocket payment ratio for medical bills, rural residency, and skilled health workers per 1000 people were the top three contributing factors of the socioeconomic inequalities in both ADL and IADL distributions. The contribution of these three variables dropped dramatically in 2018, especially for the ADL inequality. By contrast, the contribution of physical inactivity to the inequalities of both ADL and IADL distributions increased over time. Notably, the contribution of self-rated fair economic status to the ADL inequality was reversed, changing from 3.30% in 2008 to − 4.51% in 2018 (Table 2).

**Table 2:** Contribution of personal and environmental predictors to socioeconomic inequalities of ADL and IADL

Predictor	2008					2018				
	C_k_^a^	ADL		IADL		C_k_	ADL		IADL	
		Elasticity	Contribution (%)	Elasticity	Contribution (%)		Elasticity	Contribution (%)	Elasticity	Contribution (%)
**Sex** (Ref. = Male)										
Female	− 0.0071	0.0275	0.51	0.0812	0.42	−0.0091	0.0352	0.46	0.0938	0.32
**Age** (Ref. = ≤80 years)										
>80	−0.0024	0.1639	1.12	0.5588	1.06	−0.0004	0.2164	0.13	0.5574	0.09
**Marital status** (Ref. = Others)										
Living with spouse	0.0066	−0.1245	1.77	− 0.2330	0.92	0.0071	− 0.1871	1.71	− 0.2982	0.71
**Self-rated economic status** (Ref. = Very poor)										
Poor	−0.7800	− 0.0061	−1.26	− 0.0067	− 0.39	− 0.8800	− 0.0031	−2.28	− 0.0023	− 0.44
Fair	0.0564	−0.0519	3.30	−0.0595	1.05	−0.0879	−0.0791	−4.51	− 0.0545	−0.81
Rich	0.8592	−0.0091	1.56	−0.0141	0.67	0.7781	−0.0238	2.92	−0.0192	0.61
Very rich	0.9901	−0.0009	0.01	−0.0013	0.01	0.9735	−0.0028	0.08	−0.0020	0.01
**Years of schooling** (Ref. = 0)										
1–5	0.0318	−0.0085	0.10	−0.0151	0.05	0.0114	−0.0094	0.02	−0.0136	0.01
≥ 6	0.1549	−0.0043	0.17	−0.0096	0.10	0.1271	−0.0046	0.13	−0.0114	0.08
**Occupation** (Ref. = Others)										
Agriculture	−0.0217	−0.0569	−3.42	− 0.0808	−1.35	− 0.0241	− 0.0280	− 0.96	− 0.0052	−0.05
**Regular exercise** (Ref. = Yes)										
No	−0.0139	0.0023	0.09	0.3558	3.95	−0.0168	0.2310	**6.12**	0.6523	**4.49**
**Chronic disease** (Ref. = No)										
Yes	−0.0188	0.0350	0.67	0.0387	0.20	0.0088	0.0116	−0.06	0.0163	−0.02
**Residency** (Ref. = Urban)										
Rural	−0.0171	0.3145	**14.28**	0.3859	**4.87**	−0.0149	0.0545	1.08	0.0500	0.26
**Community support services** (Ref. = 0)										
≥ 1	0.0312	0.0022	−0.03	− 0.0017	0.01	0.0088	0.0002	0.01	−0.0024	0.01
**Social security program enrolment** (Ref. = 0)										
1	−0.0321	− 0.0206	− 0.59	− 0.0281	− 0.22	− 0.0451	− 0.0282	−0.59	− 0.0331	−0.18
≥ 2	0.1420	−0.0024	0.13	−0.0066	0.10	0.0666	−0.0144	0.47	−0.0198	0.17
**Living arrangement** (Ref. = With family members)										
Alone	−0.1328	0.0009	0.03	0.0012	0.01	−0.0739	0.0132	0.14	0.0051	0.01
In an institution	0.0791	−0.0306	0.08	−0.0459	0.03	0.0344	−0.0279	0.03	−0.0371	0.01
**Household members (Ref. = 0)**										
1–2	−0.0049	−0.0239	− 0.09	−0.0309	− 0.03	−0.0091	− 0.1029	−0.46	− 0.1316	−0.15
≥ 3	−0.0568	−0.0205	− 0.70	− 0.0335	− 0.32	− 0.0201	− 0.0438	− 0.23	− 0.0596	−0.08
**Access to medical care** (Ref. = Yes)										
No	−0.0340	0.0424	2.56	0.0393	0.66	−0.0118	0.1116	1.26	0.0773	0.23
**Out-of-pocket payment ratio (%)**	−0.0176	0.0076	**19.28**	0.0048	**3.38**	−0.0148	0.0081	**8.21**	0.0056	**1.48**
**Per capita income**	0.0019	−0.0001	3.08	−0.0002	1.71	0.0006	−0.0002	3.45	0.0003	−1.35
**Skilled health workers per 1000 people**	0.0083	−0.1393	**7.67**	−0.2043	**3.13**	0.0075	−0.1278	**6.26**	−0.1338	**2.16**
**Hospital beds per 1000 people**	0.0083	−0.0318	1.37	−0.0092	0.11	0.0017	−0.0625	0.59	−0.0482	0.12

^a^ C_k_: the concentration index of the predictive variables

### Decomposition of changes in inequalities

The socioeconomic inequalities of functional disability increased by 65.88 and 70.12% for the ADL and IADL distributions, respectively, over the ten-year period as measured by the CI coefficients.

The distributional and elasticity changes of self-rated economic status made the largest contribution to the changes in ADL and IADL inequalities, with a self-rated economic status as fare pushing the inequalities towards the equality line. The next contributors that had a counter-effect on the growing inequalities were rural residency, living with chronic conditions, in an institution and household members. The effects of rural residency, and chronic conditions were mainly due to their changes in elasticity rather than in distributions. By contrast, the effect of living in an institution and living with 1 or 2 household members was mainly attributable to its change in distributions (Table 3).

**Table 3:** Oaxaca-type decomposition of changes in ADL and IADL inequalities between 2008 and 2018

Predictor	ADL			IADL		
	Distributional change (Δc*η_kt_)	Elasticity change (Δη*c_kt-1_)	Contribution to change(%)	Distributional change (Δc*η_kt_)	Elasticity change (Δη*c_kt-1_)	Contribution to change(%)
**Sex** (Ref. = Male)						
Female	−0.0001	− 0.0001	2.23	− 0.0002	−0.0001	2.41
**Age** (Ref. = ≤80 years)						
>80	0.0004	−0.0001	−5.48	0.0011	0.0001	−9.72
**Marital status** (Ref. = Others)						
Living with spouse	−0.0001	−0.0004	9.06	−0.0001	− 0.0004	5.04
**Self-rated economic status** (Ref. = Very poor)						
Poor	0.0003	−0.0023	36.25	0.0002	− 0.0034	27.84
Fair	0.0114	−0.0015	**−176.43**	0.0079	0.0003	**−70.84**
Rich	0.0019	−0.0126	191.07	0.0016	−0.0044	24.56
Very rich	0.0001	−0.0019	32.76	0.0001	−0.0007	5.74
**Years of schooling** (ref. = 0)						
1–5	0.0002	0.0001	−2.91	0.0003	0.0001	−2.83
≥ 6	0.0001	0.0001	−1.45	0.0003	−0.0003	−0.33
**Occupation** (Ref. = Others)						
Agriculture	0.0001	−0.0006	**10.02**	0.0001	−0.0016	**14.16**
**Regular exercise** (Ref. = Yes)						
No	−0.0007	−0.0032	**68.74**	−0.0019	− 0.0041	**52.29**
**Chronic disease** (Ref. = No)						
Yes	0.0003	0.0004	**−13.57**	0.0003	0.0004	**−7.57**
**Residency** (Ref. = Urban)						
Rural	0.0001	0.0044	**−81.53**	0.0001	0.0057	**−50.90**
**Community support services** (Ref. = 0)						
≥ 1	0.0001	−0.0001	1.19	0.0001	0.0001	−0.28
**Social security program enrolment** (Ref. = 0)						
1	0.0004	0.0002	−10.90	0.0004	0.0002	−5.14
≥ 2	0.0011	−0.0017	11.04	0.0015	−0.0019	3.32
**Living arrangements** (Ref. = With family members)						
Alone	0.0008	−0.0016	15.29	0.0003	−0.0005	1.89
In an institution	0.0012	0.0002	**−26.08**	0.0017	0.0007	**−20.47**
**Household members** (Ref. = 0)						
1–2	0.0004	0.0004	**−14.63**	0.0006	0.0005	**−9.10**
≥ 3	−0.0016	0.0013	5.07	−0.0022	0.0015	6.13
**Access to medical care** (Ref. = Yes)						
No	0.0025	−0.0024	−2.23	0.0017	−0.0013	−3.69
**Out-of-pocket payment ratio (%) for medical bills**	0.0000	0.0001	−0.25	0.0001	0.0001	−0.01
**Per capita income**	0.0001	0.0001	0.01	0.0001	0.0001	0.01
**Skilled health workers per 1000 people**	0.0001	0.0001	−3.53	0.0001	0.0006	−6.02
**Hospital beds per 1000 people**	0.0004	−0.0003	−2.82	0.0003	−0.0003	0.05

The major contributors to the growing socioeconomic inequalities of the ADL and IADL distributions included self-rated economic status as poor or rich, physical inactivity and engagement in farming in an earlier life. While the effect of farming was mainly reflected in its change in elasticity, both distributional and elasticity changes were indicated for physical inactivity. Surprisingly, the distributional and elasticity changes in having two or more social security programs contributed to the growing socioeconomic inequalities (Table 3).

## Discussion

This study revealed that functional disability of older adults worsened over time. One potential reason can attribute to changes in lifestyle with the improvement of living standards in China. More older people lack sufficient physical activity and form a sedentary lifestyle [33]. Additionally, an increasing incidence of chronic diseases among older adults also made a positive contribution to the higher scores of ADL and IADL in 2018 [34].

More importantly, this study found there exists socioeconomic inequality in functional disability of older people in China. The poor suffered more from functional disability compared with their rich counterparts and such a gap increased by more than 60% over the period from 2008 to 2018. The growing inequality is associated with multiple personal and environmental contributing factors, with some having a positive effect while others having a negative effect.

Self-rated economic status is the largest single contributing factor to the changes of the inequalities. The distributional and elasticity changes in self-rated status as poor or rich contribute to the growth of the inequalities while those with a fare rating have a counter-effect. Previous studies showed that people with low income have limited resources to take care of their health simply because they have to spend a large proportion of income on subsistence needs [35]. By contrast, the rich invest more for improving health. Over the past few decades, there has been a widening income gap in China, despite a rapid overall growth in wealth [36]. There is a positive sign though as indicated in this study: more people reported their economic status as fare or rich whilst less reported poor.

Physical inactivity was found to be a contributor to the increased inequalities, due to both of its distributional and elasticity changes. This finding highlights the growing importance of regular exercise as well as its unequal distribution across populations in association with functional disability. Indeed, unbalanced development has become a serious issue of concern in China. This is reflected in the distribution of sporting infrastructure, such as community parks and athletic tracks, with the poor and less developed communities lagging behind [37, 38]. Empirical evidence shows that there is also a lack of recognition on the importance of physical activities among the people with low socioeconomic status [39]. These two factors may reinforce each other, contributing to the growth of inequality in functional disability.

Farming was found to be another contributor to the increased inequalities, mainly due to its elasticity change. This is concerning as the rapid distributional change resulting from the process of urbanisation may exert limited impacts, if any, on reducing the inequalities. Previous studies found that farming in China is very labour intensive, which has a long-term detrimental effect on the physical functioning of those in their later life [40, 41]. Such an effect can be accumulative without necessarily being apparent at an younger age despite serious health consequences on older persons [2, 42]. It is reasonable to expect a more profound effect of farming when the population is becoming increasingly older over time.

It is important to note that some personal and environmental factors were found to have a counter-effect, alleviating the tendency of the worsening inequalities. Two of the factors are rural residency and living with chronic condition. Their alleviating effects are mainly attributable to the elasticity changes. This means that the effect of rural residency is unlikely to be driven by the continuous acceleration of urbanisation in China [43]; instead, it is likely to be driven by the improvement of rural infrastructure and a shrinking urban-rural gap in socioeconomic development [44]. Similarly, people may have learnt to better manage chronic conditions over time [45], alleviating the trend of increased inequalities in functional disability, although living with chronic conditions by itself is associated with functional disability [46, 47].

The distributional change of living in an institution was also found to help alleviate the tendency of the worsening inequalities. This may be associated with the recent development of the aged care system in China. In the past, only older persons who did not have family support, nor enough resources to support themselves were allowed to live in the government-subsidised aged care facilities. Nowadays, more older people (or their family) pay to live in aged care facilities in order to obtain supportive services that are otherwise unavailable due to a lack of family capacity to care for older adults. Because of the shrinking family size resulting from the decades-long family planning policy, the Chinese government also offers financial incentives to encourage the development of aged care facilities [48]. It is envisaged that more older people will have to live in an institution in the future. Similarly, an increase in people who lived with one or two household members over time created a positive effect on health status due to better accessibility of family care and informal support.

This study found that the role of social security programs in reducing socioeconomic inequality of functional disability is offset by the increased number of social security programs. Social security programs are designed to protect the most vulnerable populations, helping them to access the services they need [49]. However, most social security programs in China have adopted an insurance-alike arrangement, requiring individual contributions for enrolments. This may have excluded those with low income to get access to some programs that are accessible for their wealthier counterparts, such as retirement pension. Therefore, the enjoyment of a larger number of social security programs may be an indication of increase inequality [50].

This study has several limitations. Firstly, community-level explanatory variables were limited by the availability of data although health resources indicators at the provincial level were included in data analyses. Secondly, the socioeconomic status of study participants was assessed using a self-rated indicator, instead of a synthetised index capturing multiple socioeconomic characteristics such as income, occupation and education. Although the self-rated indicator has its own advantage, we cannot exclude the possibility of differed estimates if other SES measures are adopted. Thirdly, the decomposition analysis did not intend to establish causal interpretations. The nature of the cross-sectional data prevented us from reaching causal conclusions.

## Conclusion

Functional disability in older adults increased in China over the ten-year period from 2008 to 2018, so did the socioeconomic inequality in functional disability of older people. Lower socioeconomic status is associated with higher ADL and IADL scores. Although self-rated economic status is the single most powerful predictor of the inequality of functional disability, the growing and dominant rating of older persons with a fare economic status offsets the detrimental effects of other (poor or rich) ratings on the changes in equality. Re-distribution of wealth remains to be a powerful instrument for addressing the inequality issue, but alone it is not enough. The Oaxaca decomposition analysis suggests that the enlarged inequalities in functional disability are attributable to the increasing importance of regular exercise and its distributional changes, as well as the accumulative long-term effect of farming in earlier life. However, there is evidence that the improved rural lives and increased access to aged care facilities in China may have alleviated the trend of increasing inequalities. In addition, social security initiative can alleviate the trend of increasing inequalities, although multiple social security program enrolments appear to exacerbate the increased inequalities. This does not necessarily mean that institutional care will offer a solution to the inequality problem, nor expanding social security programs would be destined to fail. They are the unique features for a rapid transitional economy like China. Policy makers need to consider intervention strategies beyond financial measures to alleviate the growing socioeconomic inequality of functional disability. These may include, but not limited to, the development of more equitable social security and aged care system.

## Supplementary Information

**Additional file 1: Table S1.** Definition and measurements of explanatory variables.

## Data Availability

The data described in this article can be freely and openly accessed at Peking University Open Research Data: http://opendata.pku.edu.cn/dataset.xhtml?persistentId=doi:10.18170/DVN/XRV2WN

## Abbreviations

CLHLS: Chinese Longitudinal Healthy Longevity Survey
IADL: Instrumental activities of daily living
ADL: Activities of daily living
CI: Concentration index
SES: Socioeconomic status
GALI: Global Activity Limitation Indicator
LMICs: Low- and middle-income countries
ICF: International Classification of Functioning, Disability and Health
SD: Standard deviation
