# Supplementary material for: Changes of inequality in functional disability of older populations in China from 2008 to 2018: a decomposition analysis
Source: BMC Geriatr. 2022 Apr 9;22:308. doi: 10.1186/s12877-022-02987-8 (PMC8994264; doi:10.1186/s12877-022-02987-8)
Supplement: Supplementary file 1 — Additional file 1: Table S1. Definition and measurements of explanatory variables. [file 12877_2022_2987_MOESM1_ESM.docx]

**Supplementary files**

**Table S1. Definition and measurements of explanatory variables**

| **Group** | **Subgroup** | **Variable name** | **Type of variable** | **Measurements** |
| --- | --- | --- | --- | --- |
| Personal factors | Demographic | Sex | Categorical:  1=Male  2=Female | Self-reported by respondents at the time of the survey |
|  |  | Age | Categorical:  1=﹤80 years  2= ≥80 years | Self-reported by respondents at the time of the survey |
|  |  | Marital status | Categorical:  1=Others  2=Married and living with spouse | Current marital status reported by respondents at the time of the survey |
|  | Socioeconomic | Self-rated economic status | Categorical:  1=Very poor  2=Poor  3=Fair  4=Rich  5=Very rich | How do you rate your economic status in comparison with others in your local area? |
|  |  | Years of schooling | Categorical:  1=0  2=1-5  3=6 or more | How many years did you attend school? |
|  |  | Occupation | Categorical:  1= Others  2= Agriculture | What was your primary occupation before the age of 60? |
|  |  | Residency | Categorical:  1=Urban  2=Rural | Current residential area of respondent |
|  | Health and health behaviours | Regular exercise | Categorical:  1=Yes  2=No | Do you exercise regularly at present? |
|  |  | Chronic disease | Categorical:  1=No  2=Yes | Are you suffering from any of the following chronic diseases: hypertension, diabetes, heart disease, stroke, cerebrovascular disease, bronchitis, emphysema, asthma, pneumonia, pulmonary tuberculosis, cataracts, glaucoma, cancer, prostate tumor, gastric or duodenal ulcer, Parkinson’s disease, bedsore, arthritis, dementia, epilepsy or others? |
| Environmental factors | Macro-environment | Income per capita at the provincial level | Continuous | Income per capita in the province where the respondent is located. The data was extracted from the China Health Statistics Yearbook for 2008 and 2018. |
|  |  | Skilled health workers per 1000 people | Continuous | Number of skilled health workers per 1,000 people in the province where the respondent is located. The data was extracted from the China Health Statistics Yearbook for 2008 and 2018. |
|  |  | Hospital beds per 1000 people | Continuous | Number of hospital beds per 1,000 people in the province where the respondent is located. The data was extracted from the China Health Statistics Yearbook for 2008 and 2018. |
|  | Meso-environment | Community support services available | Categorical:  1=0  2=1 or more | What kind of social services are available in your community (personal daily care services, home visits, psychological counselling, daily shopping, social and recreation activities, legal aid, health education, neighborhood socialising and others)? |
|  |  | Social security program enrolment | Categorical:  1=0  2=1  3=2 or above | Are you covered by any of the following social security and commercial insurances programs at present (None, retirement pension, public senior insurance, commercial senior insurance, public free medical services, medical insurance for urban employees and residents, new rural cooperative medical insurance, commercial medical insurance and others)? |
|  | Micro-environment | Living arrangement | Categorical:  1=With family members  2=Alone  3=In an institution | Co-habiting status reported by respondents. |
|  |  | Household member living together | Categorical:  1=0  2=1-2  3=3 or more | How many people are living with you? (excluding yourself). If the respondent lives alone, it will be assigned a value of 0 |
|  |  | Out-of-pocket payment ratio (%) for medical bills | Continuous | What was the total expense of your medical care last year and how much was paid by your family (yourself, spouse, children, etc.)? |
|  |  | Access to medical services | Categorical:  1=Yes  2=No | Can you get adequate medical service when you are sick? |
